# *Leucobacter musarum* subsp. *musarum* sp. nov., subsp. nov., *Leucobacter musarum* subsp. *japonicus* subsp. nov., and *Leucobacter celer* subsp. *astrifaciens* subsp. nov., three nematopathogenic bacteria isolated from *Caenorhabditis*, with an emended description of *Leucobacter celer*

**DOI:** 10.1099/ijsem.0.000523

**Published:** 2015-11

**Authors:** Laura C. Clark, Jonathan Hodgkin

**Affiliations:** Biochemistry Department, University of Oxford, South Parks Road, Oxford OX1 3QU, UK

## Abstract

Three Gram-stain-positive, irregular-rod-shaped, non-motile, non-spore-forming bacteria were isolated from nematodes collected from Santa Antao, Cabo Verde (CBX151^T^, CBX152^T^) and Kakegawa, Japan (CBX130^T^). Based on 16S rRNA gene sequence similarity, strains CBX130^T^, CBX151^T^ and CBX152^T^ were shown to belong to the genus *Leucobacter*. This affiliation was supported by chemotaxonomic data (2,4-diaminobutyric acid in the cell wall; major respiratory quinones MK-10 and MK-11; major polar lipids phosphatidylglycerol and diphosphatidylglycerol; major fatty acids anteiso-C_15 : 0_, anteiso-C_17 : 0_ and iso-C_16 : 0_). Strains CBX130^T^ and CBX152^T^ were found to share salient characteristics. Based on morphological, physiological, chemotaxonomic and biochemical analysis, strain CBX152^T^ represents a novel species of the genus *Leucobacter*, for which the name *Leucobacter musarum* sp. nov. (type strain CBX152^T^ = DSM 27160^T^ = CIP 110721^T^) is proposed. Two subspecies of *Leucobacter musarum* sp. nov. are proposed: *Leucobacter musarum* sp. nov. subsp. *musarum* subsp. nov. (type strain CBX152^T^ = DSM 27160^T^ = CIP 110721^T^) and *Leucobacter musarum* sp. nov. subsp. *japonicus* subsp. nov. (type strain CBX130^T^ = DSM 27158^T^ = CIP 110719^T^). The third novel strain, CBX151^T^, showed genetic similarities with *Leucobacter celer* NAL101^T^ indicating that these strains belong to the same species. Based on morphological, physiological, chemotaxonomic and biochemical differences it is proposed to split the species *Leucobacter celer* into two novel subspecies, *Leucobacter celer* subsp. *celer* subsp. nov. (type strain NAL101^T^ = KACC 14220^T^ = JCM 16465^T^) and *Leucobacter celer* subsp. *astrifaciens* subsp. nov. (type strain CBX151^T^ = DSM 27159^T^ = CIP 110720^T^), and to emend the description of *Leucobacter celer* Shin *et al.* 2011.

The genus *Leucobacter* was first described by Takeuchi *et al.* (1996), and at the time of writing comprises 15 species with validly published names. Members of the genus *Leucobacter* have been isolated from diverse environments including industrial wastewater (Halpern *et al.*, 2009; Kim & Lee, 2011; Morais *et al.*, 2004), foodstuffs (Shin *et al.*, 2011; Yun *et al.*, 2011) and soil environments (Behrendt *et al.*, 2008; Ue, 2011). Several strains have been isolated from nematodes (Somvanshi *et al.*, 2007; Muir & Tan, 2007; Percudani, 2013), perhaps indicating an affinity for this environment among species of the genus *Leucobacter* in general.

A diseased nematode strain of genus *Caenorhabditis* (JU1635), subsequently observed to carry surface-adherent bacteria, was collected by M-A Félix from rotting banana trunks at a location close to Pico da Cruz (coordinates 17° 6′ 0″ N 25° 01′ 45″ E), Santa Antao, Cape Verde (Republic of Cabo Verde). Infective bacteria were transferred to the N2 laboratory strain of *Caenorhabditis elegans* by culturing diseased larval JU1635 hermaphrodites on a lawn of *Escherichia coli* OP50 for 12 h at room temperature, removing these animals before egg-laying commenced, and then adding adult N2 hermaphrodites. Progeny of these hermaphrodites showed disease symptoms (tail swelling, slow growth, adherent bacteria) and the resulting infected N2 culture was propagated at 25 °C on *E. coli* OP50 lawns. Pathogenic bacteria were isolated from this culture by picking diseased larvae to bare Nematode Growth Medium (NGM) agar lawns, incubating at room temperature for 90 min to eliminate most of the OP50 bacteria, and then transferring the larvae to Luria–Bertani (LB) plates. These were incubated at 37 °C overnight (to kill the nematodes) followed by incubation at 30 °C for 2–3 days. Small (non-*E. coli*) single bacterial colonies were picked to establish cultures, one of which was able to cause nematode surface encumbrance, but not rectal swelling, when added to healthy N2 nematodes. This defined bacterial strain CBX151^T^ (also called Verde1).

The original infected N2 strain was propagated further by picking worms with severely swollen tails for more than eight generations, followed by transfers to bare NGM plates, removal of worms and incubation at 30 °C. Five slow-growing bacterial colonies were picked from these plates, three of which were found able to induce tail swelling and larval death when added to healthy N2 nematodes. One of these three defined bacterial strain CBX152^T^ (also called Verde2). Strain CBX130^T^ was isolated following a similar protocol from *Caenorhabditis elegans* JU1088 found in Kakegawa, Japan.

Sub-cultivation was done on LB medium at 30 °C for 48 h. On this medium, all strains were able to grow at 10–30 °C but not at 4 °C or 40 °C. Gram staining was performed according to Madigan *et al.* (2009). Cell morphology was observed under a Zeiss light microscope at × 1000 magnification, with cells grown at 30 °C on LB for 48 h.

The 16S rRNA gene sequence was determined using universal primers 27F and 1495R and the amplified region and flanking sequence was later confirmed by Illumina MiSeq whole-genome sequencing. Gene fragments were assembled using mega6 software (Tamura *et al.*, 2013) and sequence comparisons made using EzTaxon (Kim *et al.*, 2012). Phylogenetic relationships between strains CBX130^T^, CBX151^T^, CBX152^T^ and their closely related strains were established using mega6. Phylogenetic trees were reconstructed using the neighbour-joining (NJ), maximum-parsimony (MP) and maximum-likelihood (ML) algorithms (Fitch, 1971; Saitou & Nei, 1987; Felsenstein, 1981 using the Tamura & Nei, 1993 substitution model), each with 1000 randomly selected bootstrap replications. All treeing methods produced comparable tree topology (representative ML tree shown in Fig. 1).

**Fig. 1.**
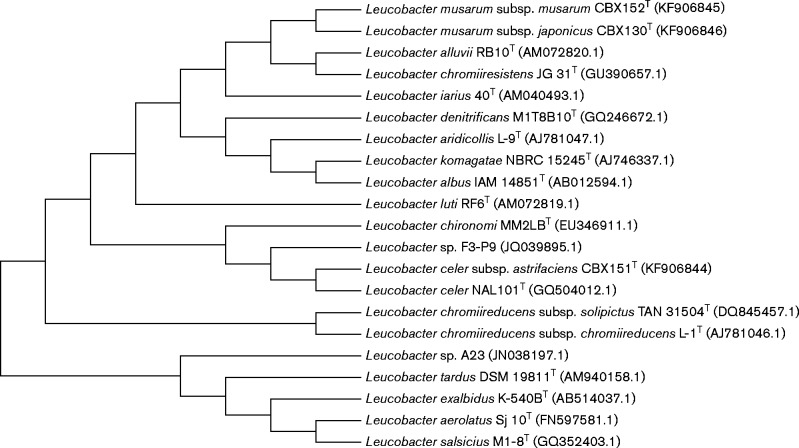
*Leucobacter* 16S rRNA gene maximum-likelihood bootstrap consensus tree. The evolutionary history was inferred by using the maximum-likelihood method based on the Tamura-Nei model. The bootstrap consensus tree inferred from 1000 replicates is taken to represent the evolutionary history of the taxa analysed. Branches corresponding to partitions reproduced in less than 50 % bootstrap replicates are collapsed. Initial tree(s) for the heuristic search were obtained by applying the neighbour-joining method to a matrix of pairwise distances estimated using the Maximum Composite Likelihood (MCL) approach. The analysis involved 21 nt sequences. All positions containing gaps and missing data were eliminated. There were 1376 positions in the final dataset. Evolutionary analyses were conducted in mega6.

The 16S rRNA gene sequences of strains CBX152^T^ and CBX130^T^ were continuous stretches of 1531 bp. Sequence similarity calculations indicated that the 16S rRNA gene sequence of strain CBX152^T^ was identical to that of strain CBX130^T^. The next closest relatives of strain CBX152^T^ were *Leucobacter chromiiresistens* JG 31^T^ (98.72 % 16S rRNA gene sequence similarity), *Leucobacter alluvii* RB10^T^ (98.52 %) and *Leucobacter komagatae* NBRC 15245^T^ (98.31 %).

The 16S rRNA gene sequence of strain CBX151^T^ was a continuous stretch of 1534 bp. Sequence similarity calculations indicated that the closest relatives of strain CBX151^T^ were *Leucobacter celer* NAL101^T^ (99.93 % 16S rRNA gene sequence similarity), *Leucobacter chironomi* MM2LB^T^ (97.98 %) and *L. chromiiresistens* JG 31^T^ (97.71 %).

Lower sequence similarities (minimum 96.57 %; maximum 98.15 % similarity for strains CBX152^T^ and CBX130^T^; minimum 96.26 %; maximum 97.39 % similarity for strain CBX151^T^) were found between the three CBX strains and all other species of the genus *Leucobacter* with validly published names.

Results of chemotaxonomic analyses are given in the species descriptions. Analysis of respiratory quinones, fatty acids, polar lipids and peptidoglycan structure for strains CBX130^T^, CBX151^T^ and CBX152^T^ was carried out by the Identification Service of the Leibniz-Institut Deutsche Sammlung von Mikroorganismen und Zellkulturen (DSMZ), Braunschweig, Germany. Briefly, methods were as follows: bacterial cells were grown in LB broth at 30 °C with shaking (210 r.p.m.) until late exponential phase and pelleted by centrifugation. Pellets were freeze-dried (for respiratory quinones, fatty acids and polar lipids) or suspended in 1 : 1 2-propanol/water (for peptidoglycan). Analysis was performed at the DSMZ using TLC for polar lipids, TLC and HPLC for respiratory quinones and gas chromatography for fatty acids. Analysis of peptidoglycan structure was performed under a variety of hydrolytic conditions using techniques including gas chromatography, TLC and mass spectrometry.

The polar lipids of strains CBX130^T^, CBX151^T^ and CBX152^T^ (Fig. S1, available in the online Supplementary Material) are consistent with those previously described in members of the genus *Leucobacter*. The cellular fatty acid profile (Table S1) is in excellent agreement with that previously reported for the genus *Leucobacter*, with anteiso-C_15 : 0_, anteiso-C_17 : 0_ and iso-C_16 : 0_ the major fatty acids in all three strains. Cell-wall amino acids are consistent with those previously reported in *Leucobacter*, with the exception that γ-aminobutyric acid (GABA) was not present in any of the strains. The quinone system (Table S2) supports affiliation of strains CBX130^T^, CBX151^T^ and CBX152^T^ to the genus *Leucobacter*, members of which typically have MK-11 and MK-10 as the major respiratory quinones (Schumann *et al.*, 2009).

Results of the physiological characterization are given in the species descriptions. Methods as described by Madigan *et al.* (2009), using colonies grown for 48 h on LB medium pH 7 and an incubation temperature of 30 °C as standard. API 50CH and 20NE test panels (bioMérieux) were used according to the manufacturer's instructions with an incubation temperature of 30 °C, and scored for up to 6 days to obtain optimum resolution between strains. Oxidase reaction was assessed using *N*,*N*-dimethyl-p-phenylenediamine discs, and catalase reaction was assessed using 3 % H_2_O_2_. Motility was assessed using the hanging-drop method. Relationship to oxygen was assessed by incubation in semi-solid thioglycollate broth. Growth medium pH was adjusted using HCl or NaOH. Results that are discriminatory between strains are summarized in Table 1. Experiments were performed at least twice and representative results are shown. *L. celer* DSM 26651^T^, *L. chromiiresistens* DSM 22788^T^ and *L. komagatae* DSM 8803^T^ were obtained from the DSMZ and used as reference strains.

**Table 1.** Physiological characteristics of type strains of selected species of the genus *Leucobacter* Strains: 1, CBX130^T^; 2, CBX152^T^; 3, CBX151^T^; 4, *L. celer* DSM 26651^T^; 5, *L. chromiiresistens* DSM 22788^T^; 6, *L. komagatae* DSM 8803^T^ (type species for the genus). All strains were negative for acid production from erythritol, d-arabinose, l-arabinose, methyl β-d-xylopyranoside, d-galactose, dulcitol, d-sorbitol, methyl α-d-mannopyranoside, methyl α-d-glucopyranoside, *N*-acetylglucosamine, amygdalin, arbutin, salicin, maltose, lactose, melibiose, sucrose, inulin, melezitose, raffinose, starch, glycogen, gentiobiose, turanose, d-fucose, l-fucose, d-arabitol, l-arabitol, potassium gluconate and potassium 2-ketogluconate. All strains were also negative for assimilation of l-arabinose, capric acid, adipic acid and phenylacetic acid, and activity of urease, arginine dihydrolase, β-galactosidase and β-glucosidase. All strains grew in the presence of apramycin (50 μg ml^− 1^) and nalidixic acid (50 μg ml^− 1^), and were sensitive to ampicillin (50 μg ml^− 1^), carbenicillin (100 μg ml^− 1^), penicillin G (100 μg ml^− 1^) and streptomycin (50 μg ml^− 1^). Experiments were performed at least twice and representative results are shown. Results for *L. komagatae* DSM 8803^T^, *L. chromiiresistenss* DSM 22788^T^ and *L. celer* DSM 26651^T^were consistent with those reported by Takeuchi *et al.* (1996), Sturm *et al.* (2011) and Shin *et al.* (2011) except: *L. komagatae* DSM 8803^T^ was negative for acid production from d-adonitol; *L. chromiiresistens* DSM 22788^T^ was positive for acid production from glycerol, d-ribose, l-rhamnose, trehalose, xylitol, d-lyxose and potassium 5-ketogluconate. These inconsistencies are likely due to a longer incubation than that used by Sturm *et al.* (2011); indeed, the results we observed at the earlier 120 h time point were consistent with theirs. +, Positive, − , negative; (+), weakly positive; na, not applicable; nd, data not available.

Characteristic	1	2	3	4	5	6
Growth in presence of (μg ml^− 1^):						
Kanamycin (50)	(+)	(+)	+	+	+	nd
Gentamicin (15)	−	(+)	(+)	+	−	nd
Erythromycin (20)	−	−	(+)	−	−	nd
Tetracycline (15)	−	(+)	(+)	+	+	−
Chloramphenicol (25)	−	−	−	−	(+)	(+)
Growth at 37 °C	−	−	+	+	+	−
Pigment production						
Constitutive	(+)	+	−	−	+	−
On light exposure	na	na	+	+	na	−
Acid production from:						
Glycerol	+	+	−	−	+	(+)
d-Ribose	−	−	+	(+)	(+)	(+)
d-Xylose	−	−	−	(+)	−	−
l-Xylose	−	−	−	+	−	−
d-Adonitol	−	−	−	(+)	−	−
d-Glucose	−	−	−	+	−	−
d-Fructose	+	−	−	+	−	−
d-Mannose	−	−	−	(+)	−	−
l-Rhamnose	+	+	+	+	(+)	−
Inositol	−	−	+	+	−	−
d-Mannitol	+	+	+	+	−	−
Aesculin	+	+	+	+	+	−
cellobiose	−	−	−	+	−	−
trehalose	−	+	−	+	+	−
Xylitol	+	+	−	−	+	−
d-Lyxose	−	(+)	−	+	(+)	−
d-Tagatose	−	(+)	−	−	−	−
Potassium 5-ketogluconate	(+)	+	−	−	(+)	−
Activity						
Protease (gelatin hydrolysis)	+	+	−	−	−	+
Oxidase reaction	−	−	+	−	−	−
Assimilation of:						
d-Glucose	+	+	−	+	+	−
d-Mannose	+	+	−	+	−	−
d-Mannitol	+	−	+	+	−	−
*N*-Acetylglucosamine	−	−	−	+	−	−
maltose	−	−	−	+	−	−
Potassium gluconate	+	−	+	+	−	−
Malic acid	(+)	+	−	−	+	−
Trisodium citrate	−	−	+	+	−	−
Respiratory quinones						
Major	MK-11	MK-10	MK-11	MK-11	MK-11	MK-11
Minor	MK-10, MK-9	MK-11, MK-9, MK-8	MK-10, MK-9	MK-9, MK-10	MK-10, MK-9, MK-8	MK-10, MK-12
Fatty acids	aiC_15 : 0_, aiC_17 : 0_, iC_16 : 0_, C_16 : 0_	aiC_15 : 0_, aiC_17 : 0_, iC_16 : 0_, C_16 : 0_	aiC_15 : 0_, aiC_17 : 0_, iC_16 : 0_, iC_15 : 0_, iC_17 : 0_	aiC_15 : 0_, aiC_17 : 0_, iC_16 : 0_, iC_15 : 0_	aiC_15 : 0_, aiC_17 : 0_, iC_16 : 0_	aiC_15 : 0_, aiC_17 : 0_, iC_16 : 0_
Polar lipids*	PG, DPG, GL, PL	PG, DPG, GL × 2, PL	PG, DPG, GL × 2	PG, DPG, GL	PG, DPG, GL, PL	PG, DPG, GL
Peptidoglycan includes:	Ala, Gly, Glu, Thr	Ala, Gly, Glu, Thr	Ala, Gly, Glu, Thr	Ala, Gly, Glu, Thr	Ala, Gly, Glu, Thr	Ala, Gly, Glu
Diamino acid†	DAB	DAB	DAB	DAB	DAB	DAB, GABA
Cross-link type	B	B	B	B	B	B
DNA G+C content (mol%)	66.76	66.77	69.09	69.22	64.23	66
Genome size (Mb)	3.59	3.44	4.15	4.16	3.37	nd

* PG, Phosphatidylglycerol; DPG, diphosphatidylglycerol; GL, unknown glycolipid; PL, unknown phospholipid.

† DAB, Diaminobutyric acid; GABA, γ-aminobutryic acid.

All strains produced a yellow pigment; CBX152^T^ and CBX130^T^ produced pigment constitutively (CBX130^T^ at a low level) while CBX151^T^ produced pigment on exposure to white light. The UV-vis absorption spectrum of methanol-extracted pigment from all three strains is consistent with that observed by Trutko *et al.* (2005) in members of the family *Microbacteriaceae* and identified as the carotenoid pigment neurosporene, also described by Muir & Tan (2007) in *Leucobacter chromiireducens* subsp. *solipictus*.

All three strains were isolated from nematodes and cause varying degrees of morbidity and mortality in *C. elegans*. Exposure to CBX151^T^ in liquid culture causes wild-type *C. elegans* N2 to stick together by their tails, forming ‘worm-stars’ (see Hodgkin *et al.*, 2013 for further description of this phenomenon). Worms exposed to *L. celer* NAL101^T^ using the same protocol do not form stars, allowing the two strains to be readily distinguished. CBX151^T^ is also lethal to the nematode *Panagrellus redivivus*, whereas *L. celer* NAL101^T^ is not.

CBX152^T^ causes a lethal infection to wild-type *C. elegans* characterized by rectal swelling, vesicle formation in the body cavity and rapid death. CBX130^T^ causes a similar tail-swelling phenotype but with comparatively little mortality.

Genomic DNA was isolated using a PowerLyser UltraClean microbial DNA isolation kit (Mobio) and sequenced using a Nextera library prep on the Illumina MiSeq platform. *De novo* contig assembly was performed using CLC Genomics Workbench. Whole-genome G+C content and genome sizes were estimated using Artemis (Rutherford *et al.*, 2000). Tetranucleotide analysis was performed using JSpecies (Richter & Rosselló-Móra, 2009). Average nucleotide identity (ANI) and genome-to-genome distance calculations (GGDC) were performed using the ANI calculator from the Kostas lab (Konstantinidis, 2007) and the GGDC tool from the DSMZ (Meier-Kolthoff *et al.*, 2013), respectively. Unless otherwise stated default parameters were used for all analyses. GGDC values were calculated using distance formula 2.

ANI and GGDC can both be used to simulate direct DNA–DNA hybridization (DDH) experiments - the ‘gold standard’ for species definition (Schumann *et al.*, 2009). An ANI of 95 ± 0.5 % corresponds to the 70 % threshold used to define species by DDH (Goris *et al.*, 2007), whereas GGDC values are expressed as a predicted DDH value and an accompanying probability that the DDH value will be above the 70 % threshold. The threshold value for tetranucleotide regression is 0.99, above which the two samples are considered to belong to the same taxon (Teeling *et al.*, 2004).

CBX151^T^ and *L. celer* NAL101^T^ had an ANI of 98.96 %, well above the 95 % threshold. This was supported by a GGDC DDH estimate of 88.5 ± 2.39 %, corresponding to a 95.23 % probability of a DDH value above 70 %. Tetranucleotide analysis gave a regression value of 0.99952, above the 0.99 threshold. Taken together, these results strongly support CBX151^T^ as a member of *L. celer*.

CBX130^T^ and CBX152^T^ had an ANI of 95.76 %, just above the 95 % threshold. GGDC predicts a DDH value of 60.70 ± 2.94 %, corresponding to a 57.75 % probability of a DDH value above 70 %. Tetranucleotide analysis gave a regression value of 0.99927, above the 0.99 threshold. Taken together, these results suggest that CBX130^T^ and CBX152^T^ belong to the same species but have undergone a degree of divergence that would justify designation as subspecies, following the examples of Brady *et al.* (2008, 2012) and Dai *et al.* (2011).

The most closely related recognised species to strains CBX152^T^ and CBX130^T^ on the basis of 16S rRNA gene sequence similarity was *L. chromiiresistens* JG 31^T^. When the *L. chromiiresistens* JG 31^T^ genome (Sturm *et al.*, 2012) was compared to that of CBX152^T^, a score of 84.84 % was obtained for ANI and 25.00 ± 2.49 % for DDH estimated by GGDC, giving a 0.02 % probability that the DDH value will be over 70 %. Similar results (84.92 %; 25.10 ± 3.51 %; 0.02 % probability) were obtained by comparing the genome of *L. chromiiresistens* JG 31^T^ with that of CBX130^T^. These results strongly support the designation of CBX130^T^ and CBX152^T^ as a novel taxon distinct from *L. chromiiresistens* JG 31^T^.

In conclusion, based on the data obtained in this study, a novel species of the genus *Leucobacter* is proposed, *Leucobacter musarum* sp. nov., which is divided into two novel subspecies: *Leucobacter musarum* sp. nov. subsp. *musarum* subsp. nov. (to accommodate strain CBX152^T^) and *Leucobacter musarum* sp. nov. subsp. *japonicus* subsp. nov. (to accommodate CBX130^T^). Furthermore, it is proposed to split the species *Leucobacter celer* into two novel subspecies, *Leucobacter celer* subsp. *celer* subsp. nov. (type strain NAL101^T^) and *Leucobacter celer* subsp. *astrifaciens* subsp. nov. (to accommodate strain CBX151^T^). An emended description of *Leucobacter celer* Shin *et al.* 2011 is also provided.

## Description of *Leucobacter musarum* sp. nov.

*Leucobacter musarum* (mus.ar′um. N.L. fem. n. *musa* banana; N.L. gen. pl. n. *musarum* of bananas).

Cells are non-motile irregular rods, and show characteristics of coryneform bacteria. Cells are Gram-stain-positive and aerobic, and contain no endogenous plasmids. When grown on LB agar, colonies are round, regular, shiny, convex, yellow. Growth occurs at 10–30 °C, pH 6–9 and in the presence of 0–5 % (w/v) NaCl. Optimal growth occurs at 30 °C, pH 7 and with 0 % NaCl. Oxidase-negative and catalase-positive. Positive for acid formation from glycerol, l-rhamnose, d-mannitol, aesculin, xylitol and potassium 5-ketogluconate; assimilation of d-glucose, d-mannose and malic acid, and production of protease. The major fatty acids are anteiso-C_15 : 0_, anteiso-C_17 : 0_ and iso-C_16 : 0_. Cell-wall amino acids are alanine, glycine, glutamine, threonine and diaminobutyric acid. Menaquinones are MK-10, MK-11 and MK-9. Polar lipids are phosphatidylglycerol, diphosphatidylglycerol, an unknown glycolipid and an unknown phospholipid.

The type strain is CBX152^T^ ( = DSM 27160^T^ = CIP 110721^T^). The DNA G+C content of the type strain is 66.77 mol%.

### Description of *Leucobacter musarum* subsp. *musarum* subsp. nov.

*Leucobacter musarum* subsp. *musarum* (mus.ar′um. N.L. fem. n. *musa* banana; N.L. gen. pl. n. *musarum* of bananas).

Characteristics are essentially as described for *Leucobacter musarum*. Additionally, positive for acid formation from trehalose, d-lyxose and d-tagatose. Contains minor amounts of MK-8 in addition to other menaquinones. Contains an unknown glycolipid in addition to polar lipids described previously. Causes a lethal infection of *Caenorhabditis elegans* characterized by rectal swelling, vesicle formation in the body cavity and rapid death.

The type strain for the subspecies is CBX152^T^ ( = DSM 27160^T^ = CIP 110721^T^).

### Description of *Leucobacter musarum* subsp. *japonicus* subsp. nov.

*Leucobacter musarum* subsp. *japonicus* (ja.po′ni.cus. N.L. adj. *japonicus* Japanese).

Characteristics are essentially as described for *Leucobacter musarum*, but differ from *Leucobacter musarum* subsp. *musarum* in the following: negative for acid production from trehalose, d-lyxose and d-tagatose, lacks MK-8, and has only one unknown glycolipid in the polar lipid profile. Positive for acid production from d-fructose and assimilation of d-mannitol and potassium gluconate. Colonies grown on LB agar are smaller and paler in colour than those of *Leucobacter musarum* subsp. *musarum*. Causes an infection of *Caenorhabditis elegans* characterized by rectal swelling and morbidity.

The type strain is CBX130^T^ ( = DSM 27158^T^ = CIP 110719^T^).

### Emended description of ***Leucobacter celer*** Shin *et al.* 2011

The characteristics are similar to those described for the genus, except that GABA is not present in the cell wall. The species contains two subspecies.

The type strain is NAL101^T^ ( = KACC 14220^T^ = JCM 16465^T^).

### Description of *Leucobacter celer* subsp. *celer* (Shin *et al.* 2011) Clark and Hodgkin 2015, subsp. nov.

The description is essentially as given by Shin *et al.* (2011). Additionally, colonies produce yellow pigment on exposure to white light. NAL101^T^ has no endogenous plasmids and an approximate genome size of 4.16 Mb. Does not cause worm-star formation in *Caenorhabditis elegans* and is not lethal to *Panagrellus redivivus*.

The type strain is NAL101^T^ ( = KACC 14220^T^ = JCM 16465^T^). The DNA G+C content of the type strain estimated from whole-genome sequence data is 69.22 mol%.

### Description of *Leucobacter celer* subsp. *astrifaciens* subsp. nov.

*Leucobacter celer astrifaciens* (as.tr.ifa′ci.ens. L. neut. n. *astrum* star; L. part. *faciens* making; N.L. part. adj. *astrifaciens* star-making).

Cells are non-motile irregular rods, and show characteristics of coryneform bacteria. Cells are Gram-stain-positive and aerobic, and contain no endogenous plasmids. When grown on LB agar, colonies are round, regular, shiny, convex, cream, producing a yellow pigment upon exposure to light. Growth occurs at 10–37 °C, pH 5–9 and in the presence of 0–2.5 % (w/v) NaCl. Optimal growth occurs at 30 °C, pH 8 and with 0 % NaCl. Oxidase- and catalase-positive. Positive for utilization of d-ribose, l-rhamnose, inositol, d-mannitol and aesculin, and assimilation of d-mannitol, potassium gluconate and trisodium citrate. The major fatty acids are anteiso-C_15 : 0_, anteiso-C_17 : 0_, iso-C_16 : 0_ and iso-C_15 : 0_. Cell-wall amino acids are alanine, glycine, glutamine, threonine and diaminobutyric acid. Menaquinones are MK-11, MK-10 and MK-9. Polar lipids are phosphatidylglycerol, diphosphatidylglycerol and two unknown glycolipids. Causes worm-star formation in *Caenorhabditis elegans* and is lethal to *Panagrellus redivivus*.

The type strain is CBX151^T^ ( = DSM 27159^T^ = CIP 110720^T^). The DNA G+C content of the type strain is 69.09 mol%.

## Supplementary Data

210 Supplementary Data
