# Supplementary material for: Leucobacter musarum subsp. musarum sp. nov., subsp. nov., Leucobacter musarum subsp. japonicus subsp. nov., and Leucobacter celer subsp. astrifaciens subsp. nov., three nematopathogenic bacteria isolated from Caenorhabditis, with an emended description of Leucobacter celer
Source: Int J Syst Evol Microbiol. 2015 Nov;65(Pt 11):3977–84. doi: 10.1099/ijsem.0.000523 (PMC4804768; doi:10.1099/ijsem.0.000523)
Supplement: Supplementary file 1 — Supplementary Data [file ijsem000523.pdf]

Figure S1. Polar lipids

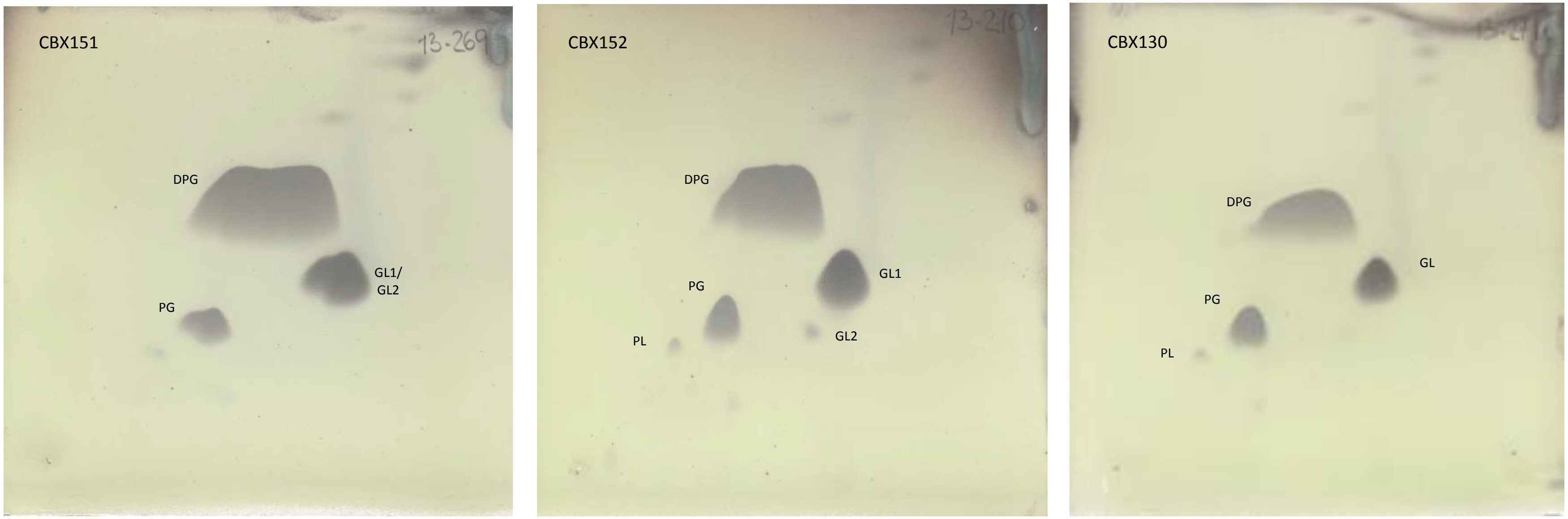

Table S1. Cellular fatty acid composition

Peak	% CBX130^T	% CBX152^T	% CBX151^T
13:0 anteiso	0.10	-	-
14:0 iso	0.45	0.26	0.28
15:0 iso	0.33	-	9.34
15:0 anteiso	45.13	42.81	41.21
15:0	-	-	-
16:0 iso	22.51	24.53	17.17
16:0	3.72	1.34	0.97
17:0 iso	0.18	-	4.39
17:0 anteiso	27.15	30.82	26.49
17:0	0.26	-	0.16
18:0 iso	0.17	0.23	-

Table S2. Menaquinone composition

Quinone	% CBX130^T	% CBX152^T	% CBX151^T
MK-8	-	3	-
MK-9	7	9	3
MK-10	28	53	26
MK-11	63	35	65
